# Calibration of ventilation/perfusion match in electrical impedance tomography: a novel method based on arterial blood pressure

**DOI:** 10.3389/fphys.2025.1545652

**Published:** 2025-03-13

**Authors:** Tixin Han, Yangchun Qin, Zhibo Zhao, Bin Yang, Xuechao Liu, Lei Li, Ziyu Wei, Liping Wei, Yifan Liu, Feng Fu

**Affiliations:** Shaanxi Key Laboratory of Bio-electromagnetic Detection and Intelligent Sensing, Military Biomedical Engineering School, Fourth Military Medical University, Xi’an, China

**Keywords:** electrical impedance tomography, pulmonary embolism, atelectasis, ventilation/perfusion match, arterial blood pressure, calibration method

## Abstract

**Introduction:**

Electrical impedance tomography (EIT) enables non-invasive, continuous, bedside evaluation of ventilation/perfusion (V/Q) match. To avoid the presence of invasive monitoring for cardiac output in relative V/Q ratio calculation, we proposed a novel calibration method based on arterial blood pressure to optimize EIT V/Q match assessments.

**Methods:**

We involved 12 mechanically ventilated piglets in three experimental phases: baseline, pulmonary embolism, and atelectasis. After a thorough measurement of EIT signals, arterial blood pressure, cardiac output, and additional physiological parameters, EIT V/Q match was evaluated using existing area limited method (ALM), cardiac output calibrated method (COCM), and our proposed novel blood pressure calibrated method (BPCM). Finally, V_D_/V_T_ and P/F ratio were calculated and correlated with V/Q match indicators derived from COCM and BPCM.

**Results:**

Arterial blood pressure waveform integration demonstrated strong correlation with cardiac output (*R* ^2^ = 0.80, p < 0.001), validating its utility for cardiac output estimation and V/Q match calibration. Both COCM and BPCM provided enhanced V/Q match region segmentation compared to ALM, yielding comprehensive diagnostic information with statistically significant differences across all three states (p < 0.05). COCM demonstrates a slightly higher correlation compared to BPCM (r = −0.63 vs. −0.52) between low ventilation index (LVI) and V_D_/V_T_, while BPCM demonstrates a slightly higher correlation compared to COCM (r = 0.49 vs. 0.44) between low perfusion index (LQI) and P/F ratio.

**Conclusion:**

This study described a novel calibration method for calculating corrected EIT-based V/Q match that utilized arterial blood pressure. Our method exhibited comparable capability in distinguishing V/Q mismatch areas compared to conventional cardiac output-based calibration techniques. With clinical data to establish a linear regression model, our method will ultimately enable us to calculate calibrated EIT V/Q match without cardiac output monitoring.

## 1 Introduction

Acute respiratory complications hold significant clinical implications in the management of patients in the Intensive Care Unit (ICU), where pulmonary embolism (PE) and atelectasis serve as two typical manifestations of ventilation/perfusion (V/Q) mismatch, significantly impacting patient prognosis (Munshi et al., 2022). Pulmonary embolism is primarily caused by deep vein thrombi obstructing the pulmonary artery, leading to pathological changes in affected areas characterized by normal ventilation but restricted perfusion (Freund et al., 2022); whereas atelectasis is commonly observed in patients who are bedridden for extended periods or post-surgery, manifesting as localized lung tissue collapse with preserved blood flow but impaired ventilation function (Bigatello and Östberg, 2021). Traditional V/Q match assessment methods, such as computed tomography (CT) and ventilation/perfusion scintigraphy (SPECT), while offering certain diagnostic value, have notable limitations: they necessitate the transfer of critically ill patients, expose them to ionizing radiation, and cannot provide continuous bedside monitoring (Lang et al., 2020; Mahaletchumy et al., 2021; Tipre et al., 2022). Therefore, developing monitoring technologies that can offer real-time, non-invasive pulmonary function assessments holds substantial clinical significance (Tomicic and Cornejo, 2019).

Electrical impedance tomography (EIT) is an emerging technology for bedside monitoring, providing real-time and continuous data that significantly enhance our understanding and management of various respiratory conditions and lung perfusion (Scaramuzzo et al., 2024). EIT also enables the quantification of V/Q matching (Reinius et al., 2015; Mauri et al., 2020; Wang et al., 2022; Scaramuzzo et al., 2024). He et al. (2021) analyzed overlapping areas between ventilation and perfusion images to calculate the percentage of match, dead space, and shunt regions, thereby facilitating the identification of three broad classifications of acute respiratory failure. Perier et al. (2020) calculated the relative V/Q ratio for each lung unit (i.e., the percentage of ventilation divided by the percentage of perfusion in each pixel), calibrated with physiological factors: cardiac output (CO) and tidal volume (V_T_). Tuffet et al. (2023) further confirmed that without calibration using V_T_/CO values, biases in the calculation of local V/Q mismatch regions would be introduced. Absolute values of ventilation distribution can be easily obtained through calibration with mechanical ventilator parameters, while invasive monitoring provides cardiac output measurements.

Consequently, Leali et al. (2024) developed a non-invasive calibration method using EIT pulsatility data, converting the relative measure of ventilation/perfusion (V/Q) mismatch into absolute values due to the correlation between EIT pulsatility and stroke volume. However, this calibration method essentially represents a self-referential approach, which substantially limits its accuracy. To further enhance the reliability and precision of the calibration, it is advisable to incorporate additional hemodynamic parameters, such as heart rate and blood pressure.

In this study, we proposed a novel calibration method based on arterial blood pressure to optimize the assessment of V/Q match via EIT. By monitoring cardiac output and arterial blood pressure across three experimental phases—baseline, pulmonary embolism, and atelectasis—in a piglet experiment, we analyzed and compared data under different conditions, thereby validating the efficacy of our method. Given that arterial blood pressure can be measured through less invasive or even non-invasive methods and has a significant correlation with stroke volume, the proposed approach is expected to become a more convenient and less traumatic bedside EIT V/Q match evaluation tool in the future, reducing patient discomfort during monitoring. This advancement offers a more humane and technologically advanced option for clinical use, signaling a new direction for the application of EIT technology in respiratory therapy.

## 2 Materials and methods

### 2.1 Experimental procedure

#### 2.1.1 Animal preparation

Twelve healthy piglets (weighing 55 ± 5 kg, 8–10 months old, of mixed sex) were selected as the subjects for this study. Prior to the experiment, the animals were fasted and water-deprived for 12 h. Anesthesia was induced via marginal ear vein injection of pentobarbital sodium (30 mg/kg), followed by tracheal intubation and connected to a ventilator (WATO EX-20 Vet, Mindray, China) for mechanical ventilation. The ventilator settings were as follows: volume-controlled ventilation (VCV) mode, tidal volume of 6–8 mL/kg, respiratory rate of 12–15 breaths per minute, inspiratory-to-expiratory ratio of 1:2, positive end-expiratory pressure (PEEP) was set manually to 0 and altered in different study phases.

#### 2.1.2 Catheter insertion and monitoring setup

A central venous catheter was inserted via the left jugular vein for fluid administration and saline contrast agent injection. A catheter was placed through the femoral artery for continuous blood pressure monitoring and blood sampling. A pulmonary artery flotation catheter (7 F, Edwards Lifesciences, United States) was inserted via the external jugular vein, and cardiac output was monitored and recorded using thermodilution (Vigilance II, Edwards Lifesciences, United States). A 16-electrode EIT belt was placed around the thorax at the level of the 4th to 5th intercostal space to monitor real-time impedance changes during ventilation and perfusion using the EIT system (PulmoVista® 500, Dräger, Lübeck, Germany).

#### 2.1.3 Experimental phases

The study consisted of three phases: 1) Baseline, 2) Pulmonary Embolism, and 3) Atelectasis. After recording all parameters under normal physiological conditions for 30-min, we inflated the balloon on the pulmonary artery flotation catheter, leading to pulmonary artery occlusion for 5–15 min. Then a 15-min equilibration was implemented to ensure that piglets did not display any significant changes in physiological status. Finally, we initially used a double-lumen endotracheal tube and performed bronchial blocking under bronchoscopy guidance. To address bilateral ventilation issues, we created a unilateral lung collapse on the non-ventilated side by setting the pneumoperitoneum machine pressure to 10 mmHg within 5 min. This procedure ensured effective lung isolation and maintained adequate ventilation of the contralateral lung. After three study phases, lungs were recruited with continuous positive airway pressure (CPAP) of 40 cm H2O for 1-min. Tidal volume was set to 5 mL kg-1 during atelectasis and increased to 8 mL kg-1 during normal phase, while PEEP was set at 0 cm H2O during pulmonary embolism and normal phase, 3 cm H2O during atelectasis. Real-time saturation of peripheral oxygen (SpO2), non-invasive blood pressure, oxygen saturation, and end-tidal carbon dioxide (ETCO2) were monitored using a multi-parameter monitor (UMEC12 Vet, Mindray, China). Artery blood pressure waveform was collected simultaneously during ventilation and saline bolus injection through femoral artery catheter using multi-channel physiological recorder (RM6240XC, Chengdu Instrument Factory, China). After each study phase, fresh arterial blood samples were collected at each phase for blood gas analysis (ABL90FLEX, Radiometer, Denmark), with partial pressure of arterial oxygen (PaO2), partial pressure of arterial carbon dioxide (PaCO2), end-tidal partial pressure of carbon dioxide (PeCO2), and potential of hydrogen (PH) acquired.

#### 2.1.4 EIT ventilation and perfusion imaging

The EIT system was configured with an excitation frequency of 80 kHz, an excitation current of 1 mA, and a data acquisition rate of 40 Hz, using a 16-electrode adjacent stimulation-measurement mode. With the subject in supine position, ventilation signals were continuously recorded for more than five respiratory cycles. EIT perfusion signals were assessed through saline bolus injection. Specifically, during an end-expiratory breath hold (≥8 s), 10 mL of 5% sodium chloride solution was injected via the central venous catheter.

Image reconstruction was performed using the GREIT algorithm (Adler et al., 2009), which generated a 32 × 32 impedance reconstruction matrix. The ventilation imaging process involved calculating the average tidal impedance changes across five breathing cycles. For perfusion imaging, we analyzed the indicator concentration dilution curve obtained from saline bolus injections (Borges et al., 2012). V and Q signals were filtered with a 128-point zero-phase finite impulse response filter (Gomez-Laberge et al., 2012). To accurately compensate for baseline drift caused by end-expiratory pauses, we identified the precise starting point of saline contrast infusion and adjusted the perfusion sequence accordingly, thereby ensuring the reliability of our EIT measurements. Finally, V and Q regions were defined as pixels higher than 10% maximum of the ventilation and perfusion maps.

### 2.2 Area limited method for V/Q match assessment

Based on the ventilation and perfusion images, we identified three distinct regions: ventilated only (A_V_), perfused only (A_Q_), and both ventilated and perfused (A_V+Q_). Using these regions, we calculated three V/Q match metrics (He et al., 2020):

(1) Match Index: The percentage of the total area is both perfused and ventilated.
(2) Deadspace Index: The percentage of the total area that is ventilated but not perfused.
(3) Shunt Index: The percentage of the total area that is perfused but not ventilated.

We refer to this assessment approach as the Area Limited Method (ALM) throughout this article. The calculation formulas are as follows (Equation 1):

$$\left\{\begin{array}{l} match\;index\left(\%\right)=\frac{A_{V+Q}}{A_{V}+A_{Q}+A_{V+Q}}\\ deadspace\;index\left(\%\right)=\frac{A_{V}}{A_{V}+A_{Q}+A_{V+Q}}\\ shunt\;index\left(\%\right)=\frac{A_{Q}}{A_{V}+A_{Q}+A_{V+Q}} \end{array}\right.$$
(1)

### 2.3 Absolute value calibration in assessing V/Q match

#### 2.3.1 Cardiac output calibrated method

First, we normalized the relative impedance changes during ventilation (∆Z_V_) and perfusion (∆Z_Q_) at each pixel. Then, we calibrated the normalized pixel values using minute ventilation (assuming that there is a 30% fixed anatomical dead space, minute ventilation can be calculated as V_T_ × respiratory rate × 0.7) for ventilation and cardiac output (CO) for perfusion. The V/Q ratio was then calculated for each pixel based on these calibrated values (Perier et al., 2020). For ease of comparison with the blood pressure integral calibration method discussed later, we refer to this approach as the Cardiac Output Calibrated Method (COCM) (Equation 2).

$${V/Q}_{\left(COCM\right)}=\frac{\frac{\Delta Z_{V\left(pixel\right)}}{\Delta Z_{V\left(total\right)}}\times \left(V_{T}\times respiratory\;rate\times 0.7\right)}{\frac{\Delta Z_{Q\left(pixel\right)}}{\Delta Z_{Q\left(total\right)}}\times CO}$$ (2)

#### 2.3.2 Blood pressure calibrated method

**FIGURE 1:**
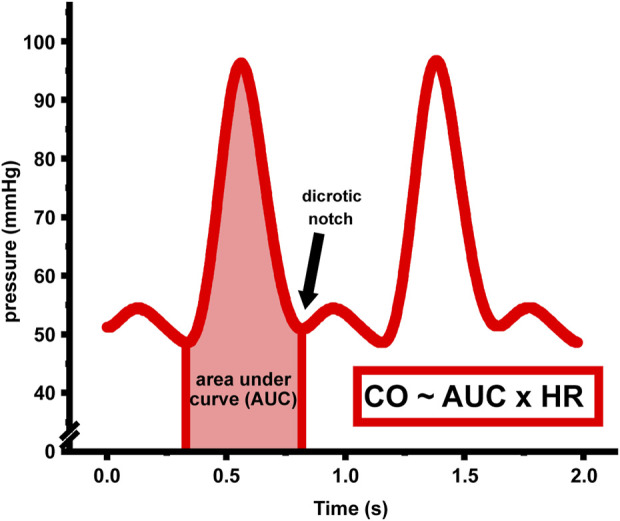
Schematic diagram of time-domain integration of arterial blood pressure waveform.

As illustrated in Figure 1, based on the research by Wesseling (1983), stroke volume (defined as blood volume ejected during each cardiac contraction) exhibits a proportional relationship to the area under curve (AUC) from the end-diastolic point to the dicrotic notch. Through this relationship, CO can be estimated by integrating the AUC of the measured parameter over a period of 1-min, which involves multiplying the AUC with the corresponding instantaneous heart rate (HR), enabling subsequent calculation of the absolute regional perfusion value.

Initially, the AUC was calculated from the arterial pressure waveform data collected during saline bolus injection using a physiological recorder. Concurrently, during the same saline bolus injection, CO measurements were obtained via thermodilution technique, and HR measurements were obtained within the arterial pressure waveform. Additionally, a linear regression analysis was conducted to establish the relationship between AUC × HR and CO values (Equation 3):

$$CO\approx \beta_{1}\times AUC\times HR+\beta_{0}$$ (3)

where β_1_ denotes the integral of arterial blood pressure, β_0_ represents the resistance parameters.

Subsequently, utilizing the improved blood pressure correction method, Blood Pressure Calibrated Method (BPCM) V/Q match calibration formula was established to optimize the perfusion calculations (Equation 4):

$${V/Q}_{\left(BPCM\right)}=\frac{\frac{\Delta Z_{V\left(pixel\right)}}{\Delta Z_{V\left(total\right)}}\times \left(V_{T}\times respiratory\;rate\times 0.7\right)}{\frac{\Delta Z_{Q\left(pixel\right)}}{\Delta Z_{Q\left(total\right)}}\times \left(\beta_{1}\times AUC\times HR+\beta_{0}\right)}$$ (4)

#### 2.3.3 Calculation of evaluation metrics

For detailed distribution analysis, the V/Q ratio of each pixel within each region underwent logarithmic transformation and was rounded to the first decimal place. The scale ranged from −1 (representing a V/Q ratio of 0.1) to 1 (representing a V/Q ratio of 10). The transformed log (V/Q) values were grouped by rounding to the nearest 10th, establishing 21 discrete intervals. To facilitate qualitative comparison, distribution curves depicting ventilation and perfusion fractions were plotted across these 21 log (V/Q) ratio intervals. The percentage area of different V/Q match regions was calculated by determining the proportion of region-specific pixels relative to the total pixel count. These calculated percentages served as quantitative evaluation metrics for assessing V/Q match distribution.

While the V/Q ratio thresholds used in this study (Pavlovsky et al., 2022) are differ from the classical MIGET standards (Wagner, 1982; Roca and Wagner, 1994). This choice was made to align with existing studies and account for the potential discrepancies between EIT pixel analysis and traditional inert gas elimination techniques.

Finally, we adapted the typical five-compartment model in a “MIGET-like” way to assess the fraction of ventilation and perfusion reaching various units: shunt (pixel V/Q ratio <0.1); low V/Q match (pixel V/Q ratio 0.1–0.8); normal V/Q match (pixel V/Q ratio 0.8–1.25); high V/Q match (pixel V/Q ratio 1.25–10); dead space (pixel V/Q ratio ≥10).

### 2.4 Comparison of V/Q match correction calculation results

The dead space index (V_D_/V_T_) and P/F ratio serve as critical parameters for evaluating pulmonary ventilation efficiency and oxygenation status. Using blood gas analysis and exhaled gas monitoring results, the dead space index (V_D_/V_T_) was calculated according to the Bohr formula (Equation 5) (Fletcher and Jonson, 1984):

$$V_{D}/V_{T}=\frac{Pa{CO}_{2}-Pe{CO}_{2}}{Pa{CO}_{2}}$$ (5)

Where:

(1) PaCO_2_ = Partial Pressure of Arterial Oxygen,
(2) PeCO_2_ = End-tidal Partial Pressure of Carbon Dioxide.

The Horowitz index (P/F ratio), calculated as the ratio of arterial oxygen partial pressure to inspired oxygen concentration (Equation 6), indicates alveolar gas exchange efficiency (Broccard, 2013):

$$P/F={PaO}_{2}/FiO_{2}$$ (6)

Where:

(1) PaO_2_ = Arterial Oxygen Partial Pressure,
(2) FiO_2_ = Inspired Oxygen Concentration.

The relationship between FiO_2_ and the P/F ratio varies depending on intrapulmonary shunting magnitude. With minimal shunting, increasing FiO_2_ elevates the P/F ratio; conversely, significant intrapulmonary shunting results in P/F ratio reduction. Thus, the P/F ratio exhibits an inverse relationship with intrapulmonary shunting, serving as a quantitative measure of shunt impact.

For comparative analysis, both low ventilation index (LVI) and low perfusion index (LQI) were calculated using V/Q match evaluation metrics derived from the COCM and BPCM. The LVI values from COCM and BPCM were summed and correlated with V_D_/V_T_, while the summed LQI values were correlated with P/F. Correlation and significance analyses were conducted to evaluate the comparative effectiveness of both methods in V/Q match assessment (Equation 7).

$$\left\{\begin{array}{l} LVI=shunt+low\;V/Q\\ LQI=deadspace+high\;V/Q \end{array}\right.$$ (7)

### 2.5 Statistical analysis

Data are presented as mean ± standard deviation (Mean ± SD) unless otherwise specified. This study employed repeated measures analysis of variance (ANOVA) with F-tests and Tukey’s *post hoc* tests to evaluate significant differences in multiple physiological parameters across three phases: baseline, pulmonary embolism, and atelectasis. Linear regression model parameters were estimated using ordinary least squares. Agreement between cardiac output and blood pressure integral calibrated values was assessed through Bland-Altman plots. Spearman correlation tests were performed to analyze relationships between: (1) V_D_/V_T_ ratios and (2) P/F ratios with LVI and LQI parameters obtained from both COCM and BPCM methods. Multiple comparison ANOVA was utilized to evaluate changes in V/Q match indices under baseline conditions and experimentally induced states (pulmonary embolism and atelectasis). Statistical significance was established at p < 0.05. All analyses were conducted using SPSS software (Version 25.0, IBM Corp., Armonk, NY, United States) and GraphPad Prism software (Version 8, GraphPad Software, San Diego, CA, United States).

## 3 Results

### 3.1 Calculation of artery blood pressure as a surrogate for cardiac output equation coefficients

**FIGURE 2:**
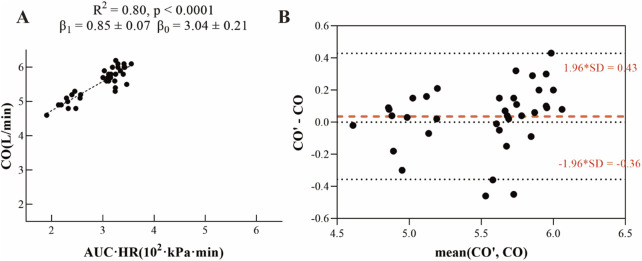
**(A)** Linear fit relationship between arterial blood pressure and cardiac output. **(B)** Bland-Altman plot comparing calculated fitted values (CO’) to measured cardiac output values (CO).

As shown in Figure 2A, data were collected from 12 experimental animals under three different conditions: baseline, pulmonary embolism, and atelectasis, yielding a total of 36 datasets. Each dataset included two key measurements: (1) the area under the arterial blood pressure curve multiplied by heart rate (AUC × HR) and (2) cardiac output (CO). A linear regression model was established to fit the data, demonstrating a significant linear correlation between AUC × HR and CO. The model yielded the following parameters. The slope β_1_ of the model was 0.85 ± 0.07, and the intercept β_0_ was 3.04 ± 0.21, with a coefficient of determination *R* ^2^ = 0.80 (p < 0.001). We also adapted linear regression separately in baseline, pulmonary embolism, and atelectasis, the results were shown in Supplementary Material, with a relatively lower *R* ^2^ value (baseline *R* ^2^ = 0.18, p = 0.165; pulmonary embolism *R* ^2^ = 0.49, p = 0.010; atelectasis *R* ^2^ = 0.38, p = 0.034), we finally decided using the equation among 36 datasets as our final equation.

Subsequently, the values of β_1_ and β_0_ were substituted into Equation 6 to develop the V/Q match parameter correction formula (Equation 8):

$${V/Q}_{\left(BPCM\right)}=\frac{\frac{\Delta Z_{V\left(pixel\right)}}{\Delta Z_{V\left(total\right)}}\times \left(V_{T}\times respiratory\;rate\times 0.7\right)}{\frac{\Delta Z_{Q\left(pixel\right)}}{\Delta Z_{Q\left(total\right)}}\times \left(0.85\times AUC\times HR+3.0\right)}$$ (8)

As illustrated in Figure 2B, the Bland-Altman analysis showed minimal outliers, demonstrating strong agreement between the calculated fitted values (CO’) from the linear regression model and the measured cardiac output values (CO). These results indicate that the linear regression model provides accurate estimates of cardiac output.

### 3.2 Schematic Diagram of EIT assessment for V/Q match

**FIGURE 3:**
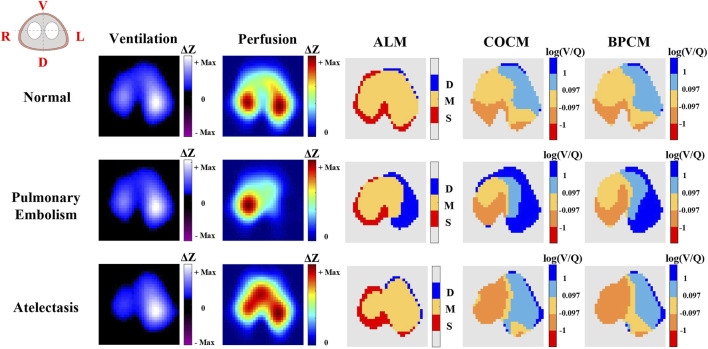
The representative results of EIT ventilation, perfusion imaging, and V/Q match obtained by different methods. In the ALM color bar, D refers to regions with ventilation only, M indicates regions with both ventilation and perfusion, and S represents regions with perfusion only. In the COCM and BPCM colorbars, the log (V/Q) was calculated to define: shunt (log (V/Q) ≤ −1); low V/Q match (−1 < log (V/Q) ≤ −0.097); normal V/Q match (−0.097 < log (V/Q) ≤ 0.097); high V/Q match (0.097 < log (V/Q) ≤ 1); dead space (1 < log (V/Q)).

Figure 3 illustrates a comparative analysis of ventilation/perfusion (V/Q) matching across three physiological states: normal conditions, pulmonary embolism, and atelectasis, utilizing three methodological approaches - ALM, COCM, and BPCM. The comparative analysis demonstrates that all three methods effectively capture the redistributional changes in V/Q match regions during the transition from normal physiological states to pathological conditions of pulmonary embolism and atelectasis. Significantly, both COCM and BPCM exhibit enhanced capability in delineating regions of low ventilation and reduced perfusion associated with pulmonary embolism and atelectasis, demonstrating superior performance compared to ALM. This visualization emphasizes the advantages of calibrated methodologies in detecting specific pathophysiological alterations within pulmonary tissue, thereby offering more precise insights into V/Q mismatch patterns.

**FIGURE 4:**
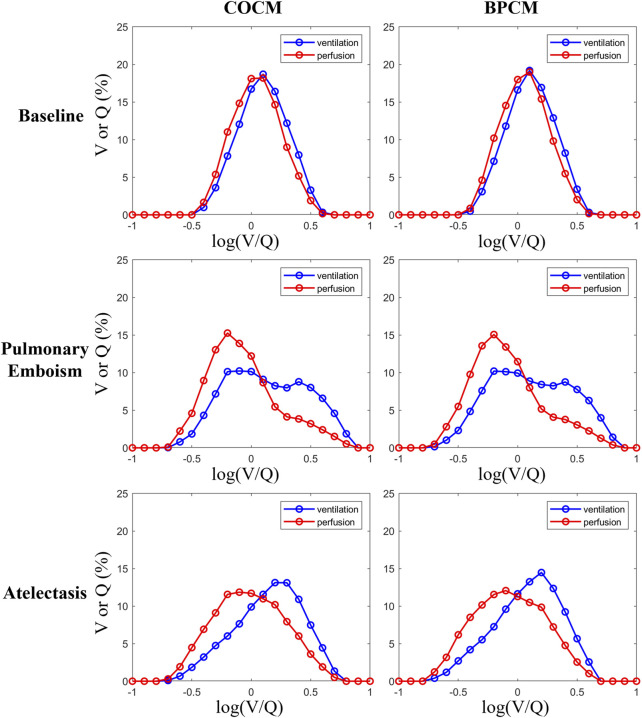
Individual data of the distribution of regional ventilation (blue curves) and perfusion (red curves) across all V/Q ratios.

Figure 4 depicts the distribution curves (bell curves) representing V/Q ratios and their corresponding ventilation and perfusion percentages obtained through COCM and BPCM analyses. The distribution patterns exhibited by these curves demonstrate remarkable similarity under both methodological approaches, indicating comparable discriminative capabilities between COCM and BPCM in distinguishing normal physiological conditions from pathological states of pulmonary embolism and atelectasis.

### 3.3 V/Q match results

#### 3.3.1 ALM results

The ALM analysis results, as presented in Table 1, demonstrate significant alterations in matching scores under pathological conditions. Specifically, the matching scores exhibited marked decreases during pulmonary embolism (64.56% ± 4.04%) and atelectasis (66.38% ± 4.44%) compared to baseline measurements (82.35% ± 3.74%). Multiple analysis of variance (ANOVA) confirmed the statistical significance of these differences (p < 0.001). Further analysis revealed distinct pathophysiological patterns: the dead space fraction demonstrated a significant increase under atelectasis conditions, elevating from 9.74% ± 3.10% at baseline to 30.22% ± 4.35% (p < 0.001). Conversely, the shunt fraction showed a substantial increase during pulmonary embolism, rising from 7.91% ± 2.72% at baseline to 27.24% ± 4.15% (p = 0.001). These quantitative findings demonstrate that the ALM-based ventilation/perfusion (V/Q) matching methodology characterizes alterations in pulmonary gas exchange during both pulmonary embolism and atelectasis conditions.

#### 3.3.2 COCM and BPCM results

**FIGURE 5:**
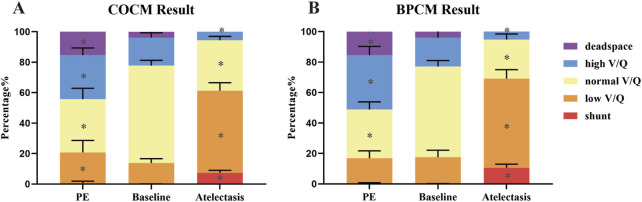
V/Q indices assessed by **(A)** COCM and **(B)** BPCM, *p < 0.05 compared to baseline.

Figure 5A, B illustrate the V/Q match results calculated using COCM and BPCM, respectively. In the COCM results, the normal V/Q ratio (normal_VQ) demonstrated a baseline measurement of 63.91% ± 3.47%, with significant reductions observed during pulmonary embolism (35.01% ± 7.14%, p < 0.001) and atelectasis (33.15% ± 2.51%, p < 0.001). The low V/Q ratio (low_VQ) increased from its baseline value of 13.79% ± 2.81% to 20.12% ± 7.90% during pulmonary embolism (p = 0.01) and demonstrated a more substantial increase to 53.94% ± 5.24% during atelectasis (p < 0.001). The high V/Q ratio (high_VQ) exhibited an increase from baseline (18.35% ± 3.19%) to 28.86% ± 4.71% during pulmonary embolism (p = 0.01) but decreased significantly to 5.58% ± 2.72% during atelectasis (p < 0.001). The shunt fraction, with a baseline of 0.03% ± 0.10%, remained statistically unchanged during pulmonary embolism (0.61% ± 1.23%, p = 0.245) but increased significantly during atelectasis (7.32% ± 1.69%, p < 0.001). The dead space fraction increased from baseline (3.92% ± 1.78%) to 15.40% ± 4.70% during pulmonary embolism (p < 0.001) and decreased to 0.00% ± 0.00% during atelectasis (p = 0.002).

In the BPCM results, the normal V/Q ratio exhibited a baseline value of 59.49% ± 3.93%, with significant reductions observed during pulmonary embolism (31.98% ± 4.99%, p < 0.001) and atelectasis (25.59% ± 3.65%, p < 0.001). The low V/Q ratio maintained stable from baseline (17.48% ± 4.61%) during pulmonary embolism (16.55% ± 4.91%, p = 0.661) but demonstrated a significant increase during atelectasis (58.57% ± 5.81%, p < 0.001). The high V/Q ratio increased from baseline (19.06% ± 6.03%) to 35.75% ± 5.70% during pulmonary embolism (p = 0.01) and decreased to 5.26% ± 2.26% during atelectasis (p < 0.001). The shunt fraction, with a baseline of 0.05% ± 0.11%, remained stable during pulmonary embolism (0.25% ± 0.51%, p = 0.716) but increased significantly during atelectasis (10.57% ± 2.35%, p < 0.001). The dead space fraction increased from baseline (3.92% ± 1.78%) to 15.46% ± 4.77% during pulmonary embolism (p < 0.001) and decreased to 0.00% ± 0.00% during atelectasis (p = 0.003).

### 3.4 Physiological correlations in COCM and BPCM results

**FIGURE 6:**
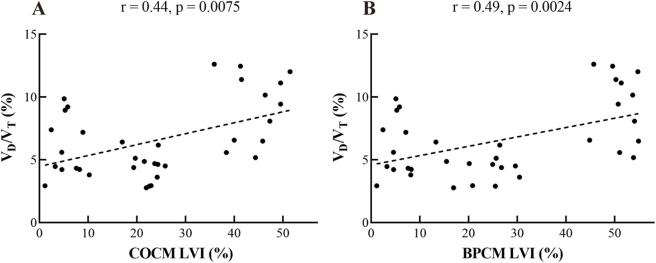
Physiological correlation between **(A)** COCM, **(B)** BPCM LVI and V_D_/V_T_.

Figure 6 presents correlation analyses between LVI and V_D_/V_T_ utilizing both COCM and BPCM. Figure 6A demonstrates that COCM analysis yields a correlation coefficient between LVI and V_D_/V_T_ of r = 0.44 (p = 0.0075), while Figure 6B reveals a slightly stronger correlation in BPCM analysis with r = 0.49 (p = 0.0024).

**FIGURE 7:**
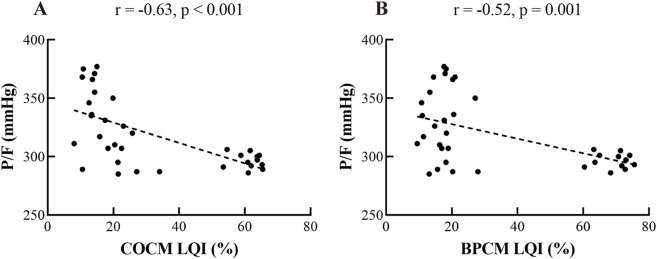
Physiological correlation between **(A)** COCM, **(B)** BPCM LQI and P/F.

Figure 7 illustrates correlation analyses between LQI and P/F ratio for both methodologies. Figure 7A demonstrates a strong negative correlation between COCM-derived LQI and P/F ratio (r = −0.63, p < 0.001). Similarly, Figure 7B reveals a significant negative correlation between BPCM-derived LQI and P/F ratio (r = −0.52, p = 0.001), though slightly less pronounced than the COCM correlation.

## 4 Discussion

Assessing pulmonary ventilation/perfusion (V/Q) mismatch is crucial for optimizing personalized mechanical ventilation therapy (Scaramuzzo et al., 2024). Existing studies have demonstrated that ventilator parameter settings, such as PEEP levels, airway plateau pressure, and alveolar compliance, exert significant influence on V/Q match dynamics (He et al., 2020; Scaramuzzo et al., 2024; Pavlovsky et al., 2022).

Traditional pixel absolute value methods for V/Q match assessment require continuous cardiac output monitoring for perfusion pixel calibration, potentially increasing the burden of patient monitoring in clinical settings. To address this limitation, we propose an innovative approach utilizing the integral of arterial blood pressure waveform (AUC × HR) as a surrogate measure for cardiac output (CO) in V/Q match index calculations. This methodological advancement derives primarily from established PICCO cardiac output measurement principles (Monnet and Teboul, 2015). Through simplification of the original mathematical relationships (Equations 3, 4) and subsequent linear coefficient fitting using experimentally derived cardiac output data (Equation 8), our results demonstrate a robust correlation between blood pressure integral and cardiac output, achieving significant statistical performance metrics (*R* ^2^ = 0.80, p < 0.001) (Figure 2). We even considered establishing linear regression models separately in three different phases (Supplementary Material). However, the *R* ^2^ values were relatively lower. It might come up with another assumption that with more data collected in different physiological status, the calibrated function can be more precise to calculate V/Q match.

Figures 3, 4 present a qualitative comparative analysis of V/Q match patterns across three physiological states: normal conditions, pulmonary embolism, and atelectasis, utilizing both COCM and BPCM. The analysis demonstrates comparable diagnostic outcomes between these methodologies. Figure 5 reveals that absolute value-based V/Q match assessments (COCM, BPCM) detect significant alterations in regional V/Q match metrics during pathological states compared to baseline measurements.

Compared to ALM, which employs a simplified three-category classification system for V/Q match (Table 1), the absolute value calculation methodologies (COCM, BPCM) provide more comprehensive diagnostic insights, and both COCM and BPCM demonstrated enhanced capability in characterizing “low ventilation areas” and “low perfusion areas”. As we conducted correlation analyses between LVI and V_D_/V_T_, the COCM demonstrates a slightly higher correlation compared to BPCM (−0.63 vs. −0.52), while between LQI and P/F ratio, the BPCM demonstrates a slightly higher correlation compared to COCM (0.49 vs. 0.44). These findings provide preliminary evidence supporting the validity of BPCM as viable alternatives to conventional cardiac output monitoring for clinical V/Q match assessment.

**TABLE 1:** Statistical analysis of ALM-Related parameters.

ALM result	Baseline	Pulmonary embolism	Atelectasis	F	p
match index (%)	82.35 ± 3.74	64.56 ± 4.04	66.38 ± 4.44	69.009	<0.001
deadspace index (%)	9.74 ± 3.10	8.21 ± 4.30	30.22 ± 4.35	115.682	<0.001
shunt index (%)	7.91 ± 2.71	27.24 ± 4.15	3.41 ± 1.71	209.845	<0.001

Finally, we believe that increasing the sample size will enhance the reliability of β, making the resulting correction factor universally applicable. Ultimately, this would eliminate the need to measure cardiac output directly; only invasive or even non-invasive arterial pressure measurements would be required.

However, several limitations of this study warrant consideration:

(1) The statistical power of our fitted equations may be constrained by the relatively modest sample size in our calibration dataset, potentially introducing discrepancies between our mathematical models and actual physiological parameters. This limitation suggests the need for validation studies with larger cohorts to enhance the robustness of our predictive equations.
(2) Current experimental protocols necessitate minimally invasive arterial cannulation for obtaining invasive arterial blood pressure measurements. While this approach provides reliable data, optimal implementation would benefit from transitioning to non-invasive, continuous, and rapid acquisition of calibration parameters.
(3) Due to the absence of mixed-venous blood samples, we were unable to directly calculate the Berggren shunt through blood gas analysis. Instead, we used the P/F ratio as an alternative measure to indirectly assess oxygenation status. However, this approach has certain limitations because the P/F ratio only reflects the oxygenation within the lungs and does not distinguish whether hypoxemia is caused by true shunting, V/Q mismatch, or other factors. Therefore, while the P/F ratio provides a useful metric for evaluating lung function, it cannot fully substitute for the precise calculation of shunt through mixed-venous blood samples.

## 5 Conclusion

We described a novel calibration method for calculating corrected EIT-based V/Q match that utilized arterial blood pressure, which is a less invasive approach compared to traditional invasive CO monitoring. To validate our ideas, we conducted animal studies with typical V/Q match scenarios: baseline, pulmonary embolism, and atelectasis. Our findings demonstrated that this novel method effectively identifies V/Q match alterations in both pulmonary embolism and atelectasis, exhibiting comparable capability in distinguishing V/Q mismatch areas compared to conventional cardiac output-based calibration techniques. We also assumed that with more data collected across various physiological states, the calibrated function can achieve higher precision in calculating V/Q matches, as it provides a better statistical foundation. To further promote our method for broader use, clinical data should be obtained that simultaneously includes cardiac output and invasive blood pressure measurements. This will allow us to establish more universal calibration equations, enabling the correction of V/Q mismatches without the need for continuous cardiac output monitoring.

## Data Availability

The original contributions presented in the study are included in the article/Supplementary Material, further inquiries can be directed to the corresponding authors.
