# Supplementary material for: Calibration of ventilation/perfusion match in electrical impedance tomography: a novel method based on arterial blood pressure
Source: Front Physiol. 2025 Mar 13;16:1545652. doi: 10.3389/fphys.2025.1545652 (PMC11966062; doi:10.3389/fphys.2025.1545652)
Supplement: Supplementary file 1 [file Table1.docx]

**Supplementary 1: Linear Regression Results Separately**

We adapted linear regression separately in baseline, pulmonary embolism, and atelectasis. In pulmonary embolism (Figure S1A), the slope β_1_ of the model was 0.73 ± 0.23, and the intercept β_0_ was 3.29 ± 0.54, with a coefficient of determination R² = 0.49 (p = 0.010). In baseline (Figure S1B), the slope β_1_ of the model was 0.71 ± 0.48, and the intercept β_0_ was 3.35 ± 1.57, with a coefficient of determination R² = 0.18 (p = 0.165). In atelectasis (Figure S1C), the slope β_1_ of the model was 0.76 ± 0.31, and the intercept β_0_ was 3.48 ± 0.99, with a coefficient of determination R² = 0.38 (p = 0.034). The parameter results of three linear regression equations were quite close to each other. Still, we believe that the equation of all these three sets will be the best one.


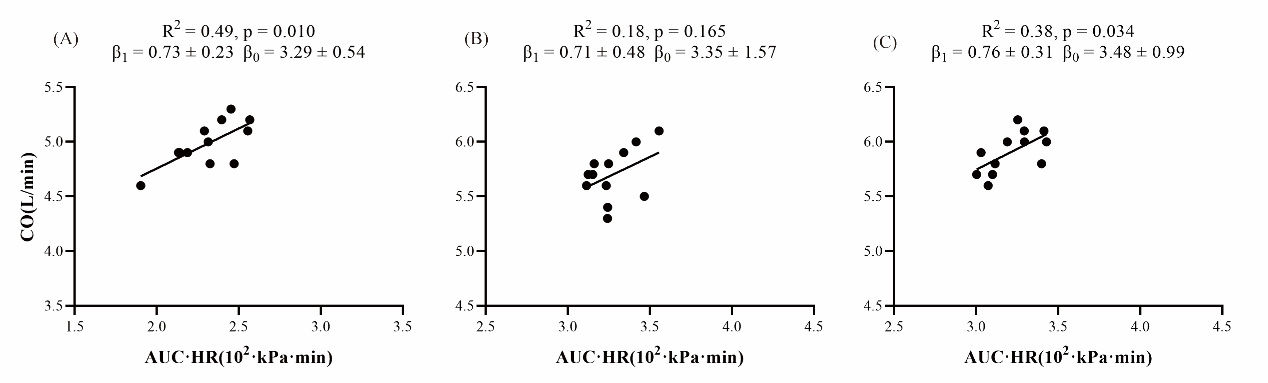


**Fig. S1.** Linear fit relationship between arterial blood pressure and cardiac output in (A) pulmonary embolism, (B) baseline, and (C) atelectasis.
