# Supplementary material for: Calibration of ventilation/perfusion match in electrical impedance tomography: a novel method based on arterial blood pressure
Source: Front Physiol. 2025 Mar 13;16:1545652. doi: 10.3389/fphys.2025.1545652 (PMC11966062; doi:10.3389/fphys.2025.1545652)
Supplement: Supplementary file 2 [file Table2.docx]

**Supplementary 2: Monitoring Results of Physiological Parameters**

As shown in Table S1, the induction of pulmonary embolism and atelectasis led to significant changes in hemodynamic and gas exchange parameters. Specifically, pulmonary embolism was characterized by a significant decrease in mean femoral arterial pressure (MAP) (p < 0.001), a significant increase in mean pulmonary arterial pressure (MPAP) (p < 0.001), a reduction in cardiac output (CO) (p < 0.001), and an increase in PaCO_2_ (p = 0.003). These alterations in hemodynamic parameters demonstrate that pulmonary embolism significantly affects pulmonary circulation and overall cardiovascular function. In contrast, atelectasis was marked by a significant decrease in PaO_2_ (p < 0.001) alongside an increase in PaCO_2_ (p = 0.001). These changes indicate that atelectasis results in compromised gas exchange efficiency and decreased oxygen delivery to tissues.

**Table S1** Hemodynamic and Gas Exchange Parameters

| **Parameters** | **Baseline** | **Pulmonary Embolism** | **Atelectasis** |
| --- | --- | --- | --- |
| MVA (l/min) | 5.27 ± 0.18 | 5.17 ± 0.21 | 3.90 ± 0.22* |
| CO (l/min) | 5.91 ± 0.19 | 4.98 ± 0.20* | 5.70 ± 0.24 |
| HR (bpm) | 72 ± 4 | 80 ± 4* | 72 ± 5 |
| MAP (mmHg) | 82.83 ± 2.76 | 66.91 ± 3.62* | 80.49 ± 4.62 |
| MPAP (mmHg) | 20.93 ± 2.09 | 30.50 ± 2.20* | 20.77 ± 2.43 |
| SpO_2_(%) | 98 ± 1 | 94 ± 1* | 88 ± 3* |
| PH | 7.40 ± 0.02 | 7.39 ± 0.02 | 7.39 ± 0.02 |
| PaO_2_ (mmHg) | 74.89 ± 3.51 | 75.83 ± 3.55 | 68.17 ± 1.48* |
| PaCO_2_ (mmHg) | 42.51 ± 1.30 | 45.17 ± 2.33* | 45.66 ± 2.05* |
| PeCO_2_ (mmHg) | 40.62 ± 1.01 | 40.95 ± 1.44 | 42.90 ± 1.93 |

*p < 0.05 compared to baseline.
